# Amperometric Glucose Biosensor Based on NiPtPd Nanozyme and Glucose Oxidase

**DOI:** 10.3390/mi17091002

**Published:** 2026-08-25

**Authors:** Asta Kausaite-Minkstimiene, Aiste Krikstaponyte, Galina Gayda, Almira Ramanaviciene

**Affiliations:** 1 NanoTechnas—Center of Nanotechnology and Materials Science, Faculty of Chemistry and Geosciences, Vilnius University, Naugarduko St. 24, LT-03225 Vilnius, Lithuania; aiste.krikstaponyte@chgf.vu.lt (A.K.); almira.ramanaviciene@chf.vu.lt (A.R.); 2 Department of Analytical Biotechnology, Institute of Cell Biology National Academy of Sciences of Ukraine (ICB NASU), Dragomanova St. 14/16, 79005 Lviv, Ukraine; galina.gayda@nas.gov.ua

**Keywords:** amperometric glucose biosensor, glucose oxidase, nanozymes, NiPtPd nanoparticles

## Abstract

Amperometric glucose biosensors remain indispensable analytical tools in clinical diagnostics due to their sensitivity, simplicity, and suitability for rapid measurements. Herein, an amperometric glucose biosensor based on immobilized glucose oxidase (GOx) and trimetallic nickel–platinum–palladium (NiPtPd) nanoparticles is reported. The NiPtPd nanoparticles deposited on a graphite rod electrode exhibited pronounced peroxidase-like activity and enabled efficient low-potential electrochemical transduction of the enzymatic reaction. The resulting biosensor displayed a linear response toward glucose in the concentration range from 0.04 to 1.58 mM. The limits of detection and quantification were determined to be 17.33 and 52.53 µM, respectively, and the sensitivity was 29.84 µA/mMcm^2^. The developed biosensor demonstrated good analytical precision, with repeatability and reproducibility characterized by relative standard deviations (RSDs) of 4.01 and 9.0%, respectively. High selectivity toward glucose, strong resistance to common electroactive interferents, and satisfactory short-term storage stability (90.78% signal retention after 14 days) were also achieved. Furthermore, preliminary glucose determination in a diluted human serum sample was demonstrated with a recovery of 101.28% and an RSD of 5.27%, indicating the potential applicability of the biosensor for analysis in diluted biological matrices. These findings demonstrate that trimetallic NiPtPd nanoparticles can serve as effective low-potential electrocatalytic transducers in enzyme–nanozyme sensing platforms.

## 1. Introduction

Research on glucose biosensing has progressed continuously since the first enzyme-based glucose electrode was introduced in 1962 [1]. A major factor supporting ongoing research in this field is the increasing global prevalence of diabetes mellitus. Consequently, precise determination of glucose concentration remains essential for both diagnosis and long-term disease management. Among the available analytical techniques, amperometric biosensors employing glucose oxidase (GOx) occupy a prominent position and are extensively utilized in clinical practice as well as in personal glucose monitoring systems [2]. Their widespread application is largely associated with favorable analytical characteristics, including high sensitivity and selectivity toward glucose, simple operation, relatively low fabrication cost, and compatibility with compact and portable device formats [3]. However, despite these advantages, GOx-based amperometric biosensors are not without limitations. Their performance can be adversely affected by the presence of electroactive interfering species, as well as by insufficient operational and storage stability, which may reduce measurement accuracy and reliability [4]. Therefore, addressing these challenges has become a key goal in the continuous development of improved biosensor platforms. In this context, nanozymes, defined as synthetic nanomaterials capable of mimicking enzyme-like catalytic activity [5], have emerged as promising components. Compared to natural enzymes, nanozymes typically exhibit improved physicochemical stability, are more cost-effective to produce, and can retain their activity over extended storage periods [6]. The combination of biological enzymes with nanozymes has opened new opportunities for the design of hybrid biosensors, where enzymatic specificity is complemented by catalytic robustness, leading to improved overall analytical performance.

Within the broad class of nanozymes, metal nanoparticle-based nanozymes composed of single or multiple metals have been studied extensively [7]. Due to their nanoscale size and unique electronic structure, they are able to effectively participate in redox processes [8], often mimicking the catalytic functions of natural enzymes such as oxidases, peroxidases, and catalases. In particular, peroxidase-like nanozymes (PO-NZs) are highly relevant for electrochemical sensing because they enable the conversion of hydrogen peroxide into an electrochemically measurable signal. Consequently, PO-NZs have been widely applied in hydrogen peroxide chemosensors [9,10] and oxidase-based amperometric biosensors [11,12]. However, despite significant progress in the development of enzyme–nanozyme-based biosensors, the integration of multimetallic nanozymes together with GOx into the design of glucose biosensors has been less extensively investigated than the integration of single-metal or bimetallic nanozymes [13,14]. Numerous studies have demonstrated that coupling GOx with nanozymes can enhance glucose sensing performance through efficient cascade catalysis and improved signal transduction [6,15]. Although these studies have highlighted the potential of enzyme–nanozyme sensing systems, further studies are needed to determine whether multimetallic nanozyme architectures can provide additional advantages in terms of electrocatalytic activity, signal transduction, and analytical selectivity in complex biological matrices. Consequently, there remains a need for sensing architectures that can provide reliable glucose detection while minimizing the influence of electroactive interferents.

One of the key requirements for practical glucose biosensors is the ability to generate a measurable analytical signal at relatively low applied potentials, where interference from electroactive compounds is reduced [4,16,17]. Monometallic and bimetallic noble metal nanoparticles have been widely investigated as electrocatalysts for hydrogen peroxide detection and oxidase-based biosensors [9,18,19]. However, multimetallic nanostructures offer the possibility of tuning catalytic properties and electrocatalytic performance through compositional design. In this context, trimetallic NiPtPd nanoparticles were selected due to the complementary catalytic properties of nickel, platinum, and palladium. Nickel can contribute to surface reactivity and at the same time reduce the amount of noble metals required [20], while platinum and palladium are well known for their electrocatalytic activity toward hydrogen peroxide reduction [9,19]. The integration of these metals into a single nanostructure was therefore considered a potential strategy for obtaining a catalytically active interface suitable for signal transduction under low-potential sensing conditions.

Although the peroxidase-like activity of NiPtPd nanoparticles cannot be unambiguously attributed to a single metallic component, previous studies have shown that platinum- and palladium-based nanomaterials exhibit intrinsic peroxidase-mimicking properties and efficiently catalyze the reduction of hydrogen peroxide [5,21,22,23]. Nickel-containing nanostructures have been reported to influence catalytic performance by modifying the electronic structure, surface adsorption characteristics, and charge transfer properties of noble metal active sites [20,21]. Consequently, the catalytic activity of NiPtPd nanoparticles may be associated with the properties of the resulting trimetallic material. Similar catalytic activity has been reported for other multimetallic nanozymes employed in electrochemical sensing applications [7,24]. Throughout this manuscript, “NiPtPd nanoparticles” refer to the physical nanomaterial, whereas “NiPtPd nanozyme” refers to its peroxidase-like catalytic function.

To the best of our knowledge, reports describing glucose biosensors based on the combined use of GOx and trimetallic NiPtPd nanozymes remain very limited. More importantly, the suitability of NiPtPd nanozymes as low-potential electrocatalytic transducers in enzyme–nanozyme glucose sensing systems has not been systematically evaluated. Furthermore, although oxidase-based biosensors are commonly interpreted in terms of hydrogen peroxide generation and detection [8,11,18], the possible influence of dissolved oxygen availability on the amperometric response of low-potential nanozyme-assisted systems has received less attention. Therefore, the primary aim of the present work was to assess the analytical performance of a GOx–NiPtPd biosensor and to investigate the electrochemical processes underlying its response.

**Scheme 1:**
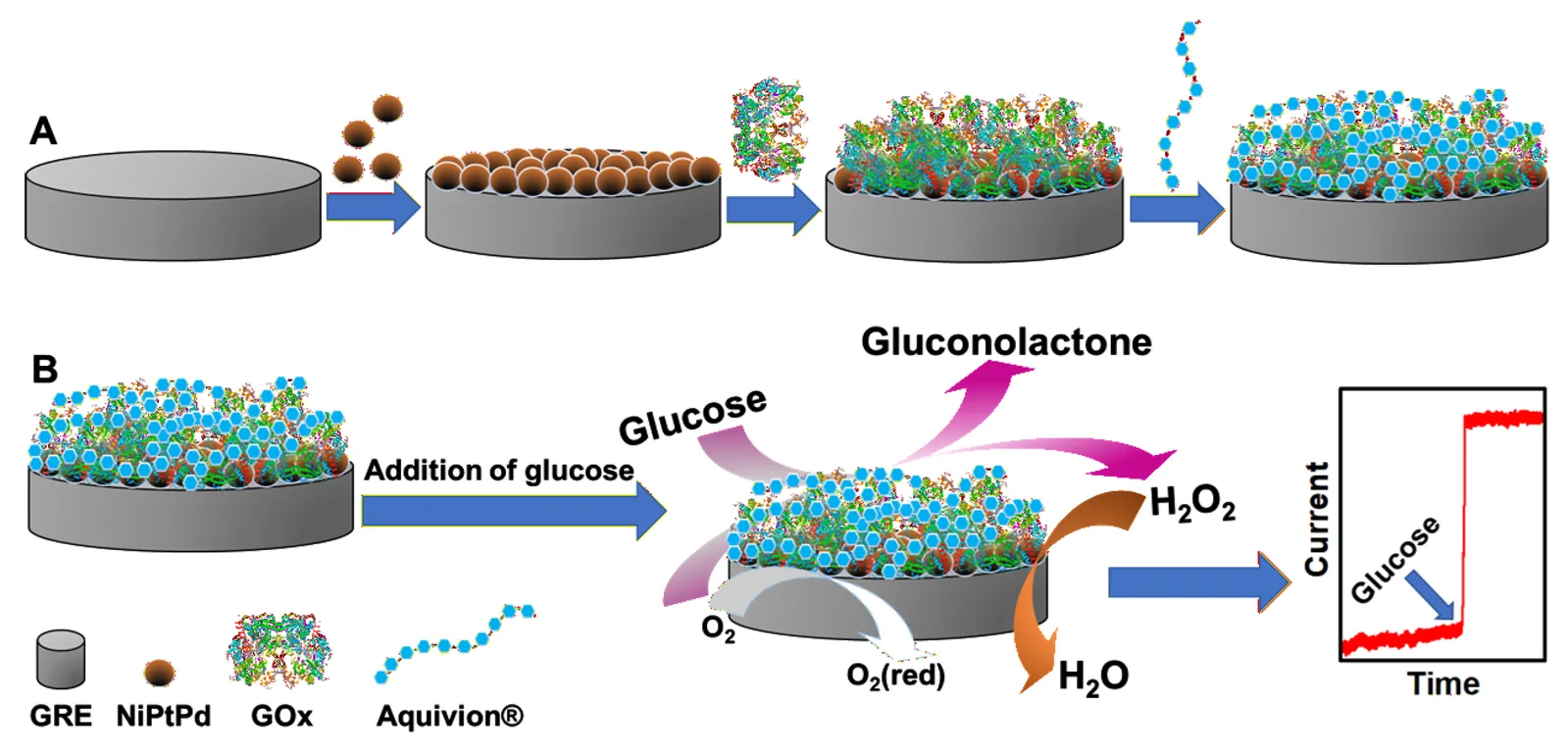
A schematic representation of the working electrode preparation (**A**) and the proposed mechanism of glucose sensing at the GR/NiPtPd/GOx/Aquivion^®^ electrode (**B**). The reduction of enzymatically generated hydrogen peroxide at the NiPtPd-modified electrode is experimentally supported, whereas the oxygen-dependent electrochemical contribution to the measured current is considered possible but was not experimentally distinguished from hydrogen peroxide reduction in the present study.

In the present study, an amperometric glucose biosensor based on the combined use of GOx and trimetallic NiPtPd nanoparticles was developed and systematically investigated. A graphite rod (GR) electrode was employed as the working electrode and modified stepwise with NiPtPd nanoparticles, GOx, and an Aquivion^®^ membrane using a layer-by-layer assembly strategy (Scheme 1A). The analytical performance of the resulting biosensor was evaluated through optimization of fabrication and operational parameters, determination of analytical characteristics, assessment of selectivity and anti-interference properties, and application to glucose determination in diluted human serum samples. The observed amperometric response was interpreted primarily in terms of the electrocatalytic reduction of enzymatically generated hydrogen peroxide, while the possible influence of dissolved oxygen availability and oxygen-dependent electrochemical processes was also considered (Scheme 1B).

## 2. Materials and Methods

### 2.1. Materials and Reagents

GOx (*Aspergillus niger*, lyophilized powder, 360 U/mg protein), sodium borohydride (NaBH_4_; ≥97%, extra pure), sodium acetate (CH_3_COONa; ≥99%, p.a., ACS, anhydrous), hexachloroplatinum (IV) acid hydrate (H_2_PtCl_6_·xH_2_O; ROTI^®^METIC 99.995%), potassium dihydrogen phosphate (KH_2_PO_4_; ≥99%, ACS reagent), disodium hydrogen phosphate dodecahydrate (Na_2_HPO_4_·12H_2_O; ≥99%, BioXtra), acetic acid (CH_3_COOH; 100%, Ph. Eur., extra pure), potassium chloride (KCl; ≥99%), sodium hydroxide (NaOH; ≥99%, beads), hydrochloric acid (HCl; 37% fuming, p.a., ACS, ISO), D(+)-xylose (C_6_H_12_O_6_; ≥99%), D(+)-mannose (C_6_H_12_O_6_; ≥99%) and D(+)-galactose (C_6_H_12_O_6_; ≥98%) were obtained from Carl Roth GmbH (Karlsruhe, Germany). Human serum (from human male AB plasma, USA origin, sterile filtered), Aquivion^®^ D98-25BS (liquid, dispersion, 25% in water, PFSA eq. wt. 980 g/mole SO_3_H, contains CF_3_ polymer chain ends as stabilizer), hydrogen peroxide (H_2_O_2_; 30% (*w*/*w*) in H_2_O, contains stabilizer), nickel (II) sulfate hexahydrate (NiSO_4_·6H_2_O; ACS reagent, ≥98%), acetaminophen (CH_3_CONHC_6_H_4_OH; ≥99.0%), palladium (II) chloride (PdCl_2_; ≥99.9%), L-ascorbic acid (C_6_H_8_O_6_; 99%), dopamine hydrochloride ((HO)_2_C_6_H_3_CH_2_CH_2_NH_2_·HCl; ≥98%) and D(+)-saccharose (C_12_H_22_O_11_; ≥99.5%) were purchased from Sigma-Aldrich Chemie GmbH (Steinheim, Germany). D(+)-glucose mono-hydrate (C_6_H_12_O_6_·H_2_O; ≥99.0%), acetylsalicylic acid (C_9_H_8_O_4_; ≥99%), diclofenac sodium (C_14_H_10_Cl_2_NNaO_2_; ≥97.5%) and norfloxacin (C_16_H_18_FN_3_O_3_) were acquired from Alfa Aesar GmbH & Co KG (Ward Hill, Massachusetts, USA). D(−)-fructose (C_6_H_12_O_6_; ≥99.5%) was obtained from Merck KGaA (Darmstadt, Germany).

### 2.2. Preparation of Solutions

Solutions, including 2.0% H_2_PtCl_6_·xH_2_O, 2.0% PdCl_2_, 0.1 M NaBH_4_, 2.0% NiSO_4_·6H_2_O, 10.0 mM NaOH, and 0.5, 1.0, 1.5, 2.0, 3.0, 4.0, and 5.0% Aquivion^®^ and buffer solutions, were prepared using ultra-high-quality (UHQ) water purified by a DEMIWA rosa 5 water purification system (WATEK, Ledeč nad Sázavou, Czech Republic). A GOx solution with a concentration of 40.0 mg/mL was prepared in a pH 6.0 acetate–phosphate-buffered solution containing 0.05 M CH_3_COONa, 0.05 M Na_2_HPO_4_, and 0.05 M KH_2_PO_4_. The acetate-phosphate buffer used for electrochemical measurements was prepared by dissolving 0.05 M CH_3_COONa, 0.05 M Na_2_HPO_4_, 0.05 M KH_2_PO_4_, and 0.1 M KCl (APB-KCl), and adjusting the pH to 5.5 or, when required, to values ranging from 3.0 to 10.0. Standard solutions of 1.0 M H_2_O_2_; 0.05 M, 0.1 M and 1.0 M glucose; 1.0 M galactose, mannose, fructose, xylose, and saccharose; 0.1 M ascorbic acid, dopamine hydrochloride, acetaminophen, diclofenac sodium and acetylsalicylic acid, and 1.5 mM norfloxacin were prepared in APB-KCl (pH 5.5). To allow for equilibration between the α- and β-anomeric forms, glucose and other saccharide solutions were prepared at least 24 h before experimentation. The GOx solution was divided into aliquots and stored at –22 °C until use, whereas all other solutions were stored at +4 °C.

### 2.3. Synthesis and Characterization of NiPtPd Nanoparticles

The NiPtPd nanoparticles were synthesized following a previously reported procedure [25], with minor modifications, and the process consisted of two sequential steps. Initially, two separate solutions, denoted as solution A and solution B, were prepared. Solution A was obtained by mixing 2.0 mL of 2.0% H_2_PtCl_6_·xH_2_O with 2.0 mL of 2.0% PdCl_2_, followed by vigorous stirring for 15 min at room temperature. Subsequently, 0.1 mL of 0.1 M NaBH_4_ was added to the mixture. Solution B was prepared by stirring 2.0 mL of 2.0% NiSO_4_·6H_2_O for 15 min at room temperature, after which 0.05 mL of 0.1 M NaBH_4_ was introduced. Afterward, solutions A and B were combined, and 0.05 mL of 10.0 mM NaOH was added. The reaction mixture was allowed to stand at room temperature without agitation for 24 h. The synthesized nanoparticles were isolated by centrifugation using an Eppendorf 5430R centrifuge at 10,000 rpm for 40 min. The collected nanoparticles were washed twice with UHQ water and stored as a 0.2 mL aqueous colloidal dispersion at +4 °C until further use.

The morphology of the NiPtPd nanoparticles was examined by a high-resolution field-emission scanning electron microscope (FE-SEM) SU-70 (Hitachi, Ibaraki, Japan) at magnifications of 50,000× and 100,000×. For FE-SEM analysis, samples were prepared by drop-casting 3 µL of NiPtPd nanoparticle colloidal dispersion onto a silicon substrate using a micropipette, followed by solvent evaporation at room temperature.

Nanoparticles synthesized according to the same protocol were previously characterized by SEM-XRM, confirming the presence of Ni, Pt and Pd in the synthesized material [25].

### 2.4. Preparation of Working Electrodes

Graphite rods (99.999% purity, 3.0 mm in diameter and 150 mm in length) obtained from Sigma-Aldrich Chemie GmbH (Taufkirchen, Germany) were cut into approximately 4 cm long segments and manually polished using successive grades of emery paper: fine (P120), very fine (P320), and ultra-fine (P2000). After polishing, the GR electrodes were rinsed with ethanol and UHQ water, dried at room temperature, and subsequently covered with a silicone tube to prevent contact between the lateral surface and the electrolyte solution.

For the preparation of working electrodes modified solely with NiPtPd nanoparticles, a defined volume (1.5, 3.0, 6.0, 9.0, 12.0, or 15.0 µL) of the NiPtPd colloidal dispersion was drop-cast onto the GR electrode surface using a micropipette, and the solvent was allowed to evaporate at room temperature (GR/NiPtPd). Subsequently, 5.0 µL of a 1.0% Aquivion^®^ solution was applied to the GR/NiPtPd electrode surface and dried at room temperature for approximately 1 h, yielding GR/NiPtPd/Aquivion^®^ electrode. In contrast, for working electrodes modified with both NiPtPd nanoparticles and GOx, aliquots (3.0, 6.0, 9.0 or 12.0 µL) of a 40.0 mg/mL GOx solution were deposited onto the GR/NiPtPd electrode surface and dried to form GR/NiPtPd/GOx electrode. This was followed by drop-casting 5.0 µL of Aquivion^®^ solution with concentrations ranging from 0.5 to 5.0%, after which the solvent was evaporated to obtain GR/NiPtPd/GOx/Aquivion^®^ electrodes. Until experiments, the prepared GR/NiPtPd/Aquivion^®^ and GR/NiPtPd/GOx/Aquivion^®^ electrodes were stored in sealed test tubes above a drop of APB-KCl (pH 6.0) at +4 °C.

### 2.5. Electrochemical Measurements

Electrochemical experiments were conducted using a PalmSens4 potentiostat (PalmSens BV, Houten, The Netherlands) controlled by PSTrace 5.10 software. Measurements were performed in a standard three-electrode configuration, employing GR/NiPtPd/Aquivion^®^ or GR/NiPtPd/GOx/Aquivion^®^ as the working electrode, an Ag/AgCl_3.0 M KCl_ as the reference, and a platinum coil as the counter electrode. All electrochemical measurements were carried out in 10.0 mL of oxygen-saturated APB-KCl buffer (pH 5.5, unless otherwise specified), inside a Faraday cage and at room temperature. Amperometric responses were recorded at a constant applied potential of +0.10 V, or at alternative potentials when required, while the electrolyte solution was continuously stirred at 350 rpm using a magnetic stirrer. Cyclic voltammetry measurements were performed by scanning the potential between –0.60 and +0.60 V at a scan rate of 0.10 V/s.

### 2.6. Interpretation of Experimental Data

The electrochemical response of the biosensor was expressed as the change in current (ΔI), calculated as the difference between the current measured after the addition of a standard glucose solution and the background current recorded in APB-KCl. Unless otherwise stated, all experiments were performed in triplicate, and the results were presented as mean values with corresponding error bars representing the standard deviation. The linear dependence of the current response on glucose concentration was established from the calibration curve by applying a linear regression model (y = ax + b) to the experimental data. From this regression, the slope (a), intercept (b), residual standard deviation (σ), and coefficient of determination (R^2^) were determined. The limit of detection (LOD) and the limit of quantification (LOQ) were calculated as 3.3σ/a and 10σ/a, respectively, where σ represents the residual standard deviation of the regression and a is the slope of the calibration curve. Sensitivity values were normalized using the geometric surface area of the graphite rod electrode.

Repeatability and reproducibility were assessed by calculating the RSD of current responses obtained from five consecutive measurements of 0.6 mM glucose using the same GR/NiPtPd/GOx/Aquivion^®^ electrode and from five independently fabricated electrodes, respectively. The RSD (%) was calculated as the ratio of the standard deviation to the mean value multiplied by 100.

Biosensor selectivity was evaluated by recording current response to different saccharides. Initially, a glucose standard solution was added to the electrochemical cell to achieve a concentration of 1.0 mM. After reaching a stable current response, standard solutions of galactose, mannose, fructose, xylose, and sucrose (each at 5.0 mM) were sequentially introduced, followed by an additional addition of 1.0 mM glucose, and the corresponding current changes were recorded.

The influence of potentially interfering electroactive species was examined similarly. Standard solutions of glucose, ascorbic acid, dopamine hydrochloride, norfloxacin, acetaminophen, diclofenac sodium, acetylsalicylic acid, and glucose (again) were successively added to the electrochemical cell to obtain final concentrations of 1.0, 0.2, 0.1, 0.003, 0.2, 0.2, 0.2, and 1.0 mM, respectively.

Storage stability was evaluated by monitoring the current response of three identically prepared GR/NiPtPd/GOx/Aquivion^®^ electrodes toward 1.0 mM glucose at regular intervals over 14 days. The recorded current responses were normalized to the initial response measured on day one and expressed as percentages, with results reported as mean values.

The analytical accuracy of the biosensor was assessed using commercial human serum samples. A defined volume of serum with a known glucose concentration, previously determined using a FreeStyle Optium glucometer (ART16648 Rev. B 05/10), was added to the electrochemical cell. The glucose concentration was then calculated from the biosensor current response using a linear equation derived from the calibration curve obtained with standard glucose solutions. Recovery (%) was determined as the ratio of the measured glucose concentration to the added concentration multiplied by 100.

## 3. Results and Discussion

### 3.1. Characterization of Morphology and Peroxidase-like Activity of NiPtPd Nanoparticles

**Figure 1:**
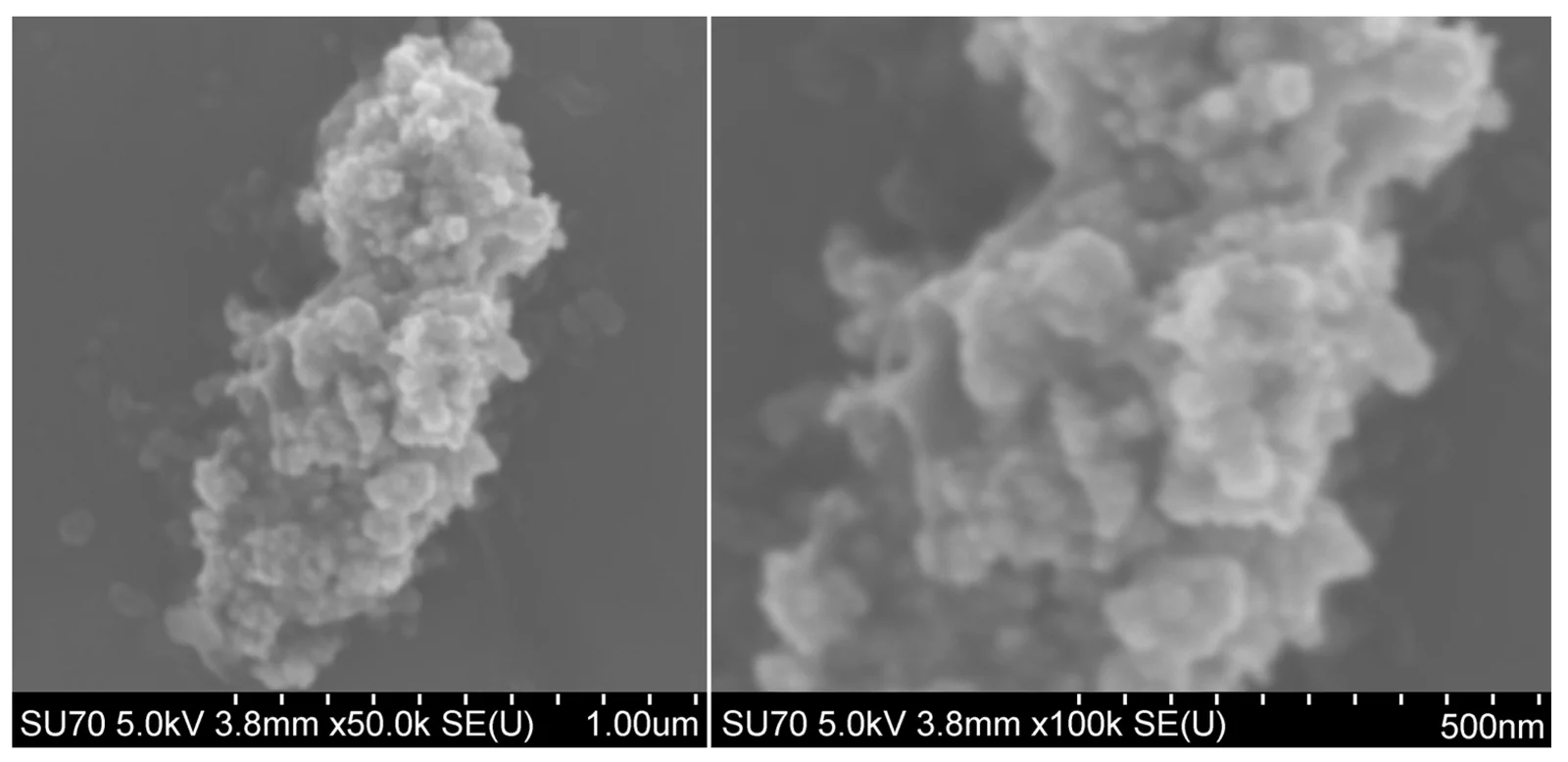
FE-SEM images of NiPtPd nanoparticles at 50,000× and 100,000× magnification.

It has been reported that the enzyme-like catalytic behavior of nanozymes is significantly affected by their physical properties, particularly particle size and shape [21]. Previous studies have shown that particle size [26] and morphology [27] can influence the catalytic performance of PO-NZs. Therefore, the morphology of the synthesized NiPtPd nanoparticles was investigated using FE-SEM. As shown in Figure 1, the synthesized material consists of interconnected coral-like aggregates rather than well-dispersed individual nanoparticles. The FE-SEM images indicate the formation of hierarchical three-dimensional structures composed of smaller nanoscale features; however, the dimensions of the primary particles cannot be reliably determined from these images alone. Therefore, no conclusions regarding the size distribution or specific surface area of the material can be drawn from the present FE-SEM analysis. Nevertheless, the observed porous aggregate morphology may facilitate electrolyte accessibility and contribute to the electrocatalytic behavior of the material. It should be noted that the characterization performed in the present study was limited to FE-SEM morphology analysis. The elemental composition of nanoparticles synthesized according to the same protocol was previously confirmed by SEM-XRM analysis [25], demonstrating the presence of Ni, Pt, and Pd. However, particle size distribution, crystallinity, crystal structure, and surface chemical composition were not determined in the present work and therefore remain unknown.

**Figure 2:**
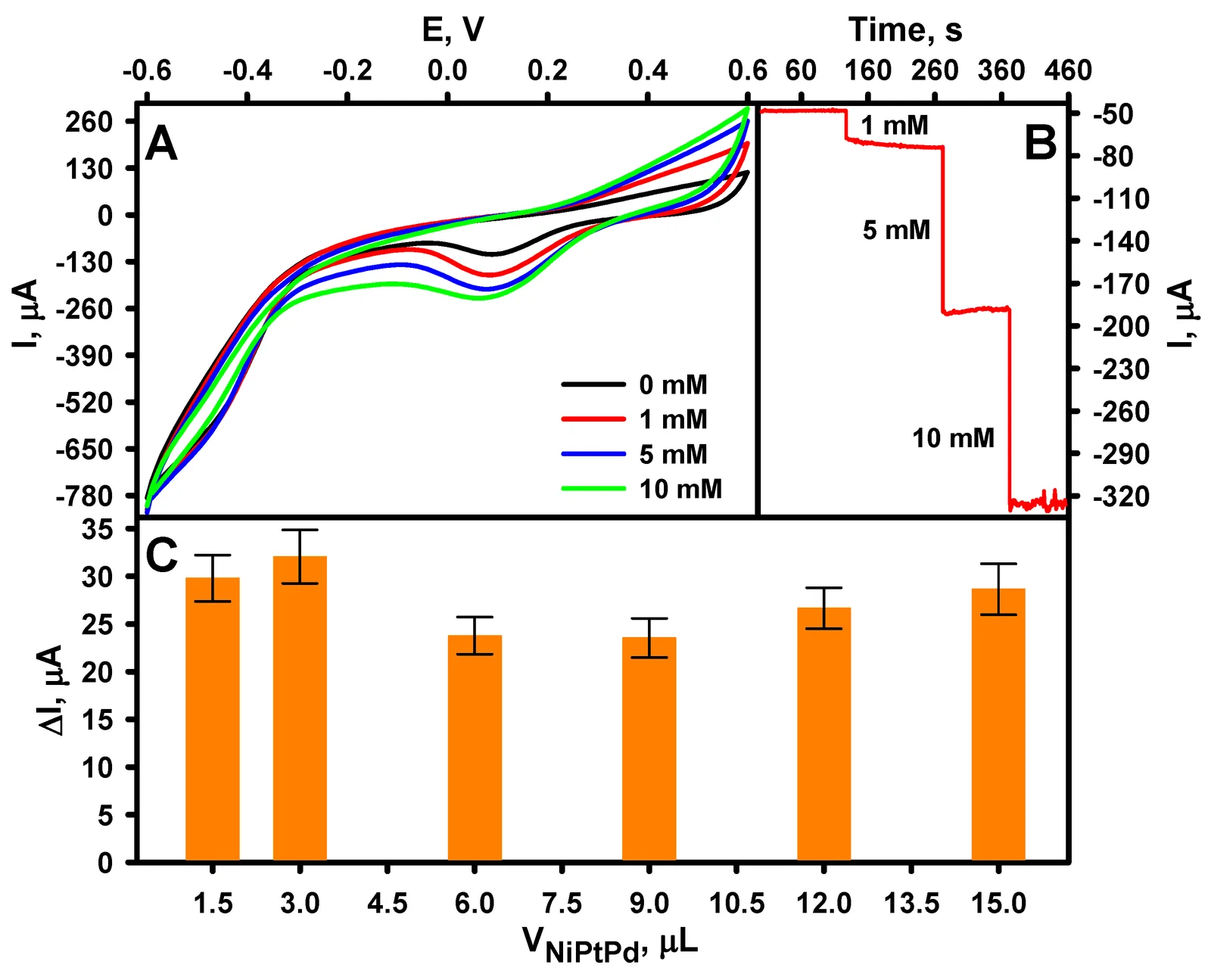
Cycling voltammograms (**A**) and amperogram (**B**) of the GR/NiPtPd/Aquivion^®^ electrode, and the influence of NiPtPd loading on the current response of the GR/NiPtPd/Aquivion^®^ electrode to hydrogen peroxide (**C**). GR/NiPtPd/Aquivion^®^ preparation conditions: 6.0 µL of NiPtPd (**A**,**B**); 5.0 µL of 1.0% Aquivion^®^ (**A**–**C**). Measurement conditions: APB-KCl (pH 6.0); 1.0 mM of H_2_O_2_ (**C**); +0.10 V applied potential vs. Ag/AgCl_3.0 M KCl_ (**B**,**C**); 0.10 V/s scan rate (**A**). Error bars represent the standard deviation of three independently prepared electrodes (n = 3).

The electrocatalytic activity of NiPtPd nanoparticles toward hydrogen peroxide reduction was investigated using cyclic voltammetry and amperometry. As shown in Figure 2A, the addition of hydrogen peroxide to the electrochemical cell resulted in a pronounced increase in cathodic current, indicating accelerated electron transfer during the reduction process. The cyclic voltammograms exhibited a well-defined cathodic peak with a maximum at approximately +0.09 V. A similar peak was also observed in the absence of hydrogen peroxide, indicating the presence of a background electrochemical process at the NiPtPd-modified electrode surface. Upon addition of hydrogen peroxide, the peak current increased with increasing hydrogen peroxide concentration, confirming the electrocatalytic activity of the GR/NiPtPd/Aquivion^®^ electrode toward hydrogen peroxide reduction.

The electrocatalytic behavior was further evaluated by amperometry at a constant potential of +0.10 V. Successive additions of hydrogen peroxide produced stepwise increases in cathodic current (Figure 2B), demonstrating a clear concentration-dependent response. These results indicate that NiPtPd nanoparticles efficiently catalyze the electroreduction of hydrogen peroxide and are therefore suitable as signal-transducing elements in the proposed GOx-based biosensor. Since hydrogen peroxide is the natural substrate of peroxidases, its electrocatalytic reduction demonstrates that the NiPtPd nanoparticles exhibit peroxidase-like catalytic activity. According to the commonly accepted definition of nanozymes as nanomaterials capable of mimicking the catalytic activity of natural enzymes, the synthesized NiPtPd nanoparticles can therefore be classified as PO-NZs.

The influence of NiPtPd nanoparticle loading on the electrochemical response of the electrode was systematically evaluated. As illustrated in Figure 2C, the GR/NiPtPd/Aquivion^®^ electrode prepared using 3.0 µL of the NiPtPd nanoparticle dispersion produced the highest current response toward hydrogen peroxide reduction. Increasing the NiPtPd loading beyond this value resulted in a decrease in the measured current. Such behavior may be associated with partial nanoparticle aggregation, reduced accessibility of catalytically active sites, and/or limitations in mass transport within thicker nanoparticle layers [28]. Based on these results, a volume of 3.0 µL of NiPtPd nanoparticle dispersion was selected for subsequent experiments because it provided the highest amperometric response under the investigated conditions.

### 3.2. Electrochemical Response of GR/NiPtPd/GOx/Aquivion^®^ Electrode Toward Glucose

**Figure 3:**
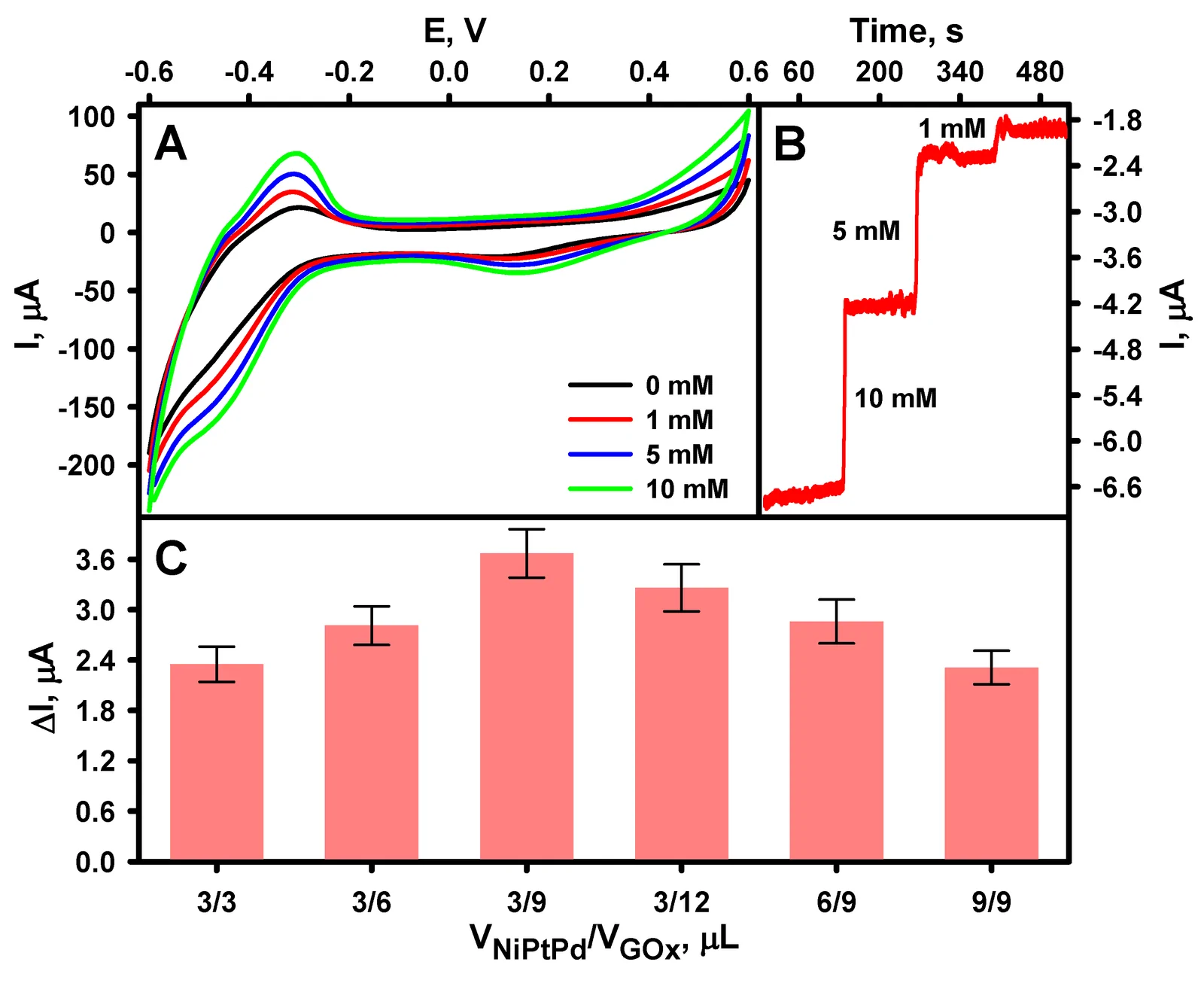
Cycling voltammograms (**A**) and amperogram (**B**) of the GR/NiPtPd/GOx/Aquivion^®^ electrode, and the influence of NiPtPd and GOx loading on the current response of the GR/NiPtPd/GOx/Aquivion^®^ electrode to glucose (**C**). GR/NiPtPd/GOx/Aquivion^®^ preparation conditions: 3.0 µL of NiPtPd; 9.0 µL of GOx (**A**,**B**); 5.0 µL of 1.0% Aquivion^®^ (**A**–**C**). Measurement conditions: APB-KCl (pH 6.0); 5.0 mM of glucose (**C**); +0.10 V applied potential vs. Ag/AgCl_3.0 M KCl_ (**B**,**C**); 0.10 V/s scan rate (**A**). Error bars represent the standard deviation of three independently prepared electrodes (n = 3).

After confirming the ability of NiPtPd nanoparticles to catalyze hydrogen peroxide reduction, their performance in the complete GOx-based sensing system was investigated. As shown in Figure 3A, cyclic voltammograms recorded for the GR/NiPtPd/GOx/Aquivion^®^ electrode exhibited a cathodic peak with a maximum at approximately +0.12 V. The magnitude of this peak increased with increasing glucose concentration, consistent with an increased availability of enzymatically generated hydrogen peroxide at the electrode interface. Amperometric measurements performed at a constant potential of +0.10 V also revealed a concentration-dependent response toward glucose (Figure 3B). Control amperometric experiments performed with the GR/NiPtPd/Aquivion^®^ electrode in the absence of GOx showed no detectable response to glucose additions, confirming that the observed glucose-dependent signal is associated with the presence of the enzyme layer. Furthermore, in contrast to the increase in cathodic current observed in cyclic voltammetry, successive additions of glucose produced a shift in the current toward more positive values. This indicates that the amperometric response of the biosensor cannot be interpreted solely in terms of hydrogen peroxide reduction and suggests that additional electrochemical processes may contribute to the measured response under the selected operating conditions.

Similar behavior has been reported for amperometric biosensors employing oxidoreductases in combination with PO-NZs [18,25,29,30,31], including our previously reported glucose biosensor based on bimetallic platinum–cobalt (PtCo) nanoparticles and GOx [32]. In that study, the electrochemical response of the GOx–PtCo system was investigated under oxygen-saturated and deoxygenated conditions, demonstrating that dissolved oxygen availability can influence the observed electrochemical response. In the present NiPtPd-based system, the opposite trends observed in cyclic voltammetry and amperometry after glucose addition are consistent with the possible involvement of oxygen-dependent processes in the measured response. The sensing mechanism of the proposed biosensor can therefore be interpreted primarily in terms of GOx-catalyzed glucose oxidation followed by electrochemical reduction of the generated hydrogen peroxide at the NiPtPd-modified electrode. The electrocatalytic activity of NiPtPd toward hydrogen peroxide reduction was directly demonstrated by the electrochemical experiments performed in the absence of glucose. At the same time, GOx catalyzes the oxidation of glucose using dissolved oxygen as the electron acceptor [33], thereby decreasing its availability for electrochemical reduction at the electrode surface. Consequently, changes in dissolved oxygen concentration may influence the measured current by altering the contribution of oxygen reduction. However, the individual contributions of hydrogen peroxide reduction and oxygen-dependent electrochemical processes were not experimentally distinguished in the present NiPtPd-based system. Therefore, oxygen depletion is considered here as a possible factor affecting the overall amperometric response rather than as a quantitatively established contribution to the analytical signal.

### 3.3. Optimization of GR/NiPtPd/GOx/Aquivion^®^ Preparation Conditions

Previous studies have demonstrated that the performance of amperometric biosensors based on enzyme–nanozyme systems is influenced not only by the individual loadings of each component on the electrode surface, but also by their relative proportion [34,35]. Figure 3C illustrates the dependence of the biosensor’s amperometric response on the loading amounts of NiPtPd nanoparticles and GOx at a fixed glucose concentration of 5.0 mM. As shown, the amperometric signal increased with increasing enzyme loading, and the highest response was obtained with the GR/NiPtPd/GOx/Aquivion^®^ electrode prepared using 3.0 µL of NiPtPd colloidal dispersion and 9.0 µL of a 40.0 mg/mL GOx solution. This behavior can be attributed to enhanced enzymatic oxidation of glucose at higher GOx loadings, leading to a stronger electrochemical response of the biosensor. At higher enzyme loadings, a decrease in the amperometric response was observed, likely due to hindered mass transport and restricted diffusion within the enzyme-rich recognition layer [36]. In contrast, increasing the amount of NiPtPd nanoparticles on the GR/NiPtPd/GOx/Aquivion^®^ electrode surface resulted in a decrease in the magnitude of the biosensor response (Figure 3C). This trend correlates well with the results obtained for the GR/NiPtPd/Aquivion^®^ electrode (Figure 2C). Although NiPtPd nanoparticles exhibit high electrocatalytic activity toward hydrogen peroxide reduction, excessive nanoparticle loading does not further enhance the amperometric response under biosensing conditions. This effect may be attributed to nanoparticle aggregation and mass transfer limitations associated with higher particle loadings. Therefore, while the overall biosensor response is governed primarily by the enzymatic oxidation of glucose and the resulting electrochemical processes occurring at the electrode interface, the presence of NiPtPd nanoparticles remains essential because they provide an efficient low-potential electrocatalytic interface through which the enzymatic reaction is transduced into a measurable amperometric signal. Considering the obtained results, 3.0 µL of NiPtPd colloidal dispersion and 9.0 µL of a 40.0 mg/mL GOx solution were selected for further experiments.

**Figure 4:**
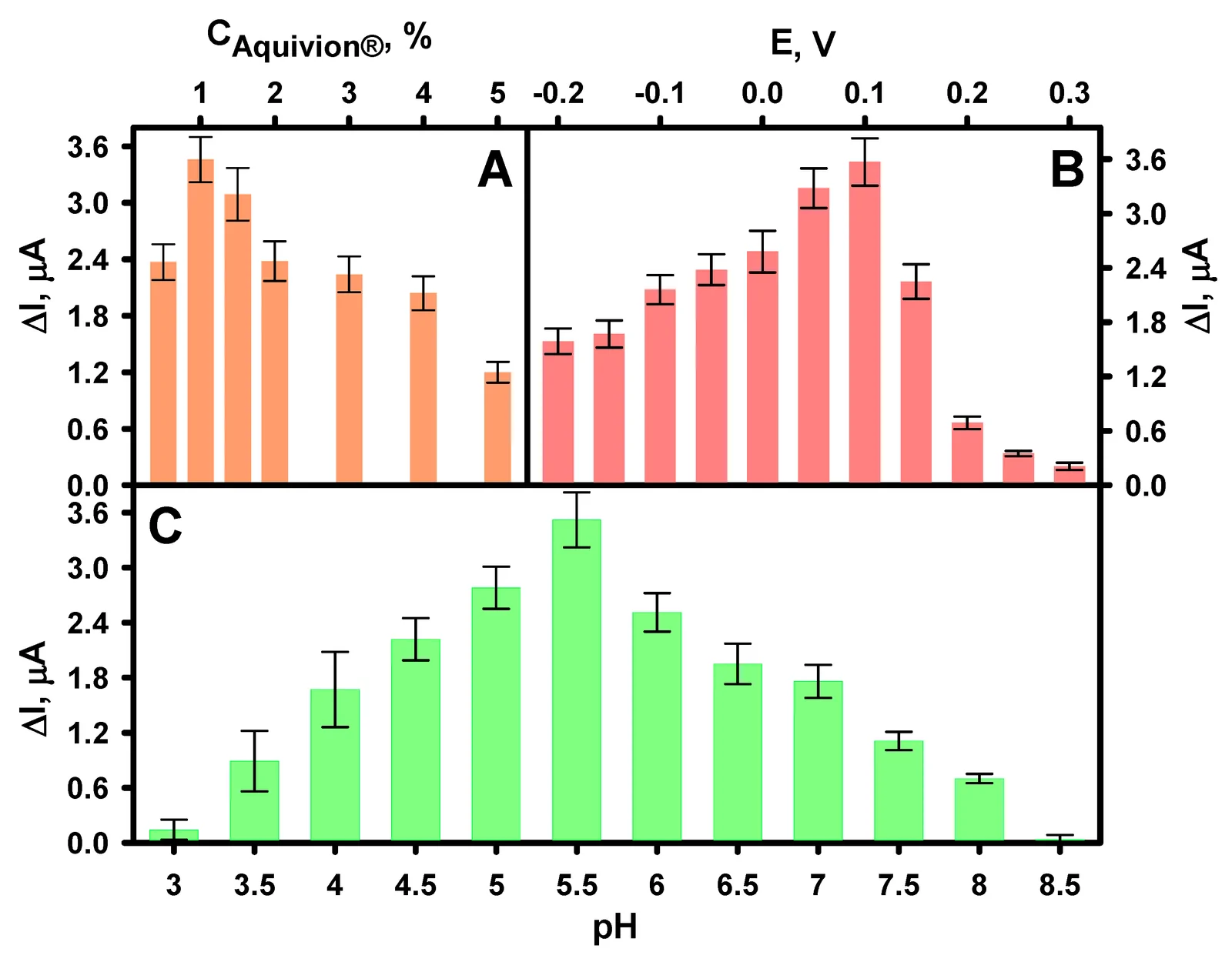
Influence of Aquivion^®^ concentration (**A**), applied potential (**B**) and APB-KCl buffer pH (**C**) on current response of GR/NiPtPd/GOx/Aquivion^®^ electrodes. GR/NiPtPd/GOx/Aquivion^®^ preparation conditions: 3.0 µL of NiPtPd; 9.0 µL of GOx; 5.0 µL of 1.0% Aquivion^®^ (**B**,**C**). Measurement conditions: APB-KCl (pH 6.0) (**A**); APB-KCl (pH 5.5) (**B**); 5.0 mM of glucose; +0.10 V applied potential vs. Ag/AgCl_3.0 M KCl_ (**A**,**C**). Error bars represent standard deviation of three independently prepared electrodes (n = 3).

The influence of Aquivion^®^ concentration on the generated biosensor signal was also investigated. As shown in Figure 4A, the highest amperometric response was obtained using a 1.0% Aquivion^®^ solution for the preparation of the GR/NiPtPd/GOx/Aquivion^®^ electrode. At lower Aquivion^®^ concentrations, the polymer layer may be insufficient to ensure efficient retention and homogeneous distribution of NiPtPd nanoparticles and GOx on the electrode surface, resulting in a lower amperometric response. In contrast, increasing the Aquivion^®^ concentration above 1.0% likely leads to the formation of a thicker polymer film, which can hinder mass transport of glucose, oxygen, and enzymatically generated hydrogen peroxide within the sensing layer. Similar diffusion limitations have previously been reported for thicker perfluorosulfonic acid coatings, leading to decreased biosensor responses [37,38,39]. Consequently, a 1.0% Aquivion^®^ concentration provides an optimal balance between effective immobilization of the sensing components and efficient mass transport, resulting in the highest biosensor response.

### 3.4. Optimization of Biosensor Operation Conditions

According to the proposed sensing mechanism, the amperometric response of the biosensor is primarily associated with electrochemical processes occurring at the GR/NiPtPd/GOx/Aquivion^®^ electrode surface following the GOx-catalyzed oxidation of glucose. These processes include the electrocatalytic reduction of enzymatically generated hydrogen peroxide, as demonstrated by the electrochemical response of the NiPtPd-modified electrode toward hydrogen peroxide. In addition, changes in dissolved oxygen availability resulting from the GOx-catalyzed reaction may influence the measured current through changes in the contribution of oxygen reduction at the electrode surface. However, the individual contributions of hydrogen peroxide reduction and oxygen depletion were not experimentally distinguished in the present study. Therefore, the magnitude of the measured response is considered to reflect primarily the enzymatic generation of electroactive species and their subsequent electrochemical processes at the modified electrode interface. Both enzymatic and electrochemical factors can influence the magnitude and direction of the measured current response. The pH of the buffer solution strongly affects the activity of glucose oxidase as well as the electrochemical behavior of the sensing interface and must be optimized to achieve maximum response, as demonstrated in enzyme [40,41] and enzyme–nanozyme-based [11,42,43] biosensing systems. Similarly, the applied potential determines the extent of the electrochemical processes contributing to signal generation and therefore directly affects biosensor sensitivity and selectivity. Operating potential has been identified as a key parameter for maximizing analytical response while minimizing interference in nanozyme-assisted electrochemical sensors [12,44]. Therefore, optimization of both pH and applied potential is essential for achieving the best analytical performance of the biosensor.

Figure 4B shows the relationship between the biosensor amperometric response and the applied potential. The response increased as the potential was shifted from −200 to +100 mV, reaching a maximum at +100 mV, and subsequently decreased at higher potentials up to +300 mV. These results demonstrate that the applied potential strongly influences the electrochemical processes responsible for signal generation and therefore affects the overall analytical response of the biosensor. Based on the obtained results, an applied potential of +100 mV was selected as optimal because it provided the highest amperometric response. Furthermore, operation at such a low potential is advantageous from an analytical perspective, since many electroactive compounds commonly present in biological samples exhibit limited electrochemical activity under these conditions. Consequently, low-potential operation is expected to improve signal selectivity and reduce susceptibility to interference. It should be noted that the selected operating potential (+0.10 V) is consistent with the potential region where the electrocatalytic reduction of hydrogen peroxide occurs, as indicated by the cathodic peak observed in cyclic voltammetry (Figure 3A).

The influence of the pH of the APB-KCl buffer solution on the amperometric response of the biosensor was investigated in the pH range from 3.0 to 8.5. The response increased with increasing pH up to 5.5 and then gradually decreased at higher pH values (Figure 4C). This behavior is consistent with previous studies demonstrating that GOx exhibits high catalytic activity in mildly acidic to near-neutral media, typically within the pH range of 5.5–7.0 [33,45,46]. In addition, the catalytic properties of metal nanoparticle-based nanozymes are known to depend on pH, with peroxidase-like activity often being favored under acidic conditions [22,23,24,47]. Therefore, the observed pH dependence of the biosensor response likely reflects the combined influence of enzyme activity and nanozyme electrocatalytic performance. Based on the obtained results, pH 5.5 was selected as the optimal pH because it provided the highest amperometric response.

### 3.5. Evaluation of the Analytical Performance of the Biosensor

**Figure 5:**
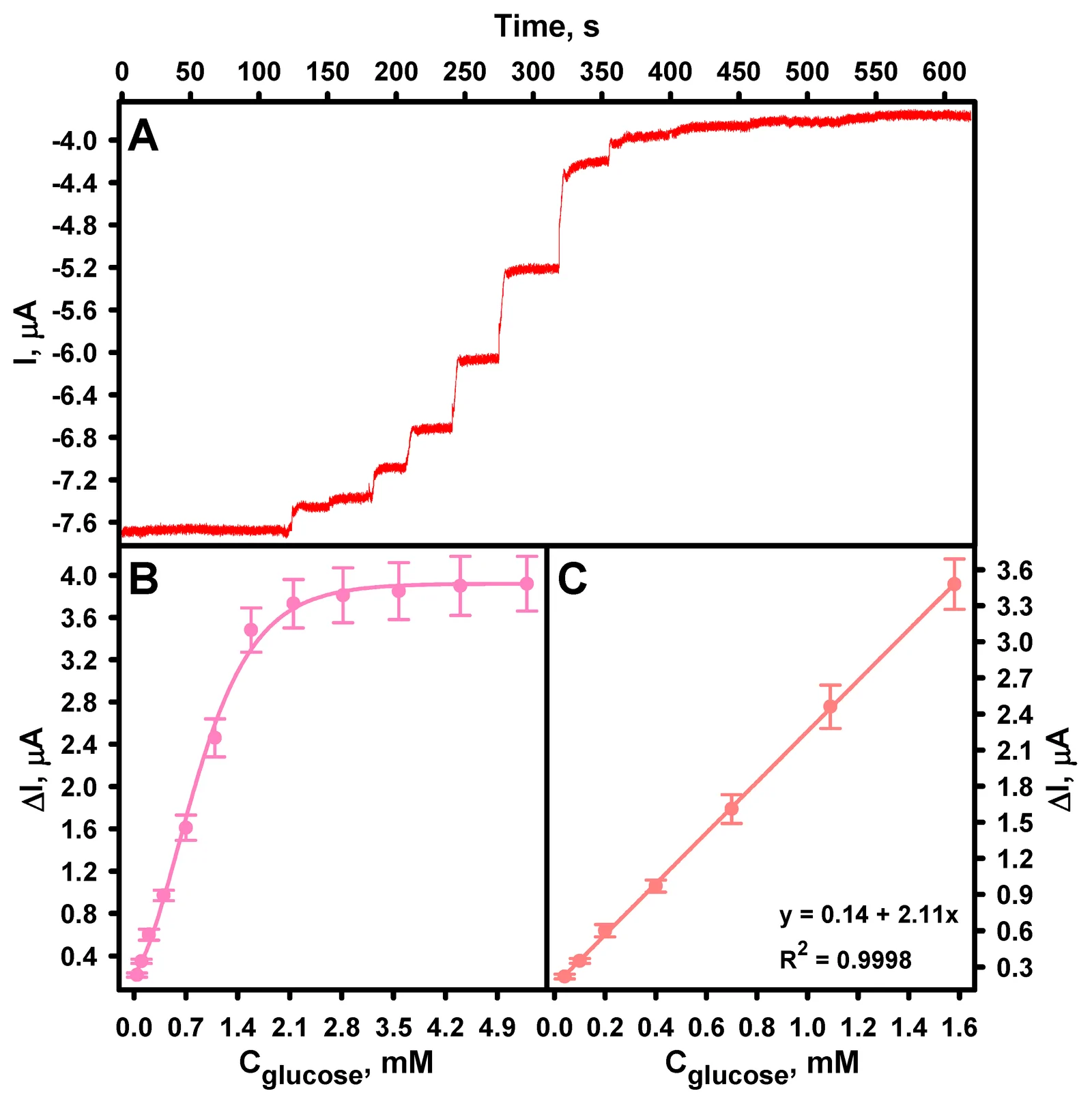
The dependence of the biosensor current response on glucose concentration (**A**,**B**) and its calibration curve (**C**). GR/NiPtPd/GOx/Aquivion^®^ preparation conditions: 3.0 µL of NiPtPd; 9.0 µL of GOx; 5.0 µL of 1.0% Aquivion^®^. Measurement conditions: APB-KCl (pH 5.5); +0.10 V applied potential vs. Ag/AgCl_3.0 M KCl_. Error bars represent the standard deviation of three replicate measurements (n = 3).

Following optimization of GR/NiPtPd/GOx/Aquivion^®^ electrode fabrication and the biosensor operating conditions, its analytical performance toward glucose was evaluated. The amperometric response exhibited a characteristic hyperbolic dependence on glucose concentration over the range of 0.04–5.3 mM, indicating saturation behavior at higher concentrations (Figure 5A,B). A linear relationship between current response and glucose concentration was observed in the range of 0.04–1.58 mM (Figure 5C), with an excellent correlation coefficient (R^2^ = 0.9998). The biosensor exhibited an LOD of 17.33 µM and a LOQ of 52.53 µM. The slope of the calibration curve was 2.11 ± 0.01 μA/mM, corresponding to a sensitivity of 29.84 μA/mM cm^2^ after normalization to the geometric surface area of the graphite rod electrode.

The analytical signal is generated by GOx-catalyzed oxidation of glucose and the subsequent electrochemical processes occurring at the NiPtPd-modified electrode surface. As discussed in Section 3.2, the measured amperometric response is likely influenced not only by the electroreduction of enzymatically generated hydrogen peroxide but also by changes in dissolved oxygen concentration during the enzymatic reaction. Nevertheless, the response remained proportional to glucose concentration within the linear range, enabling reliable quantitative determination of glucose.

The analytical characteristics of the proposed biosensor are comparable to those reported for previously described enzyme–nanozyme-based amperometric glucose biosensors (Table 1). The linear range of the developed biosensor (0.04–1.58 mM) is narrower than that reported for some glucose biosensors; however, similar working ranges are commonly observed for enzyme–nanozyme sensing systems employing peroxidase-like nanomaterials as electrocatalytic transducers. This linear range does not fully cover glucose concentrations typically encountered in undiluted human serum or whole blood samples. Therefore, dilution of biological samples is generally required when glucose concentrations exceed the upper limit of the calibration range. Accordingly, the present biosensor is more suitable for glucose determination in diluted biological samples than for direct analysis of undiluted serum. Future optimization of enzyme loading, immobilization strategy, and sensing layer architecture may help extend the linear range and reduce the need for sample dilution. Although the LOD is not the lowest among reported systems, the developed biosensor enables glucose determination at a relatively low applied potential (+0.10 V), thereby reducing susceptibility to interference from electroactive compounds and maintaining reliable analytical performance in complex biological matrices.

Reliable evaluation of repeatability and reproducibility is essential for assessing the practical applicability of electrochemical glucose biosensors [16,17]. Therefore, both parameters were investigated for the developed GR/NiPtPd/GOx/Aquivion^®^ biosensor. Repeatability was evaluated using a single electrode prepared under optimized conditions by recording amperometric responses toward 0.6 mM glucose in five consecutive measurements performed under identical conditions on the same day. The obtained RSD of 4.01% (Table 2) indicates good measurement precision. Meanwhile, reproducibility was assessed using five independently fabricated GR/NiPtPd/GOx/Aquivion^®^ electrodes, each tested toward 0.6 mM under identical conditions on the same day. An RSD value of 9.0% (Table 2) was obtained, demonstrating satisfactory electrode-to-electrode reproducibility. Although the variability observed in the reproducibility study was higher than that obtained for repeatability, it remained below 10%, which is generally considered acceptable for analytical applications. The increased variation most likely originates from minor differences in electrode fabrication, including variations in the loading and distribution of NiPtPd nanoparticles and GOx within the sensing layer. The satisfactory repeatability obtained from five consecutive measurements performed using the same electrode indicates that the developed biosensor can be reused over a limited number of measurement cycles while maintaining good measurement precision. However, long-term operational stability under an extended number of repeated measurements was not investigated in the present study and therefore the maximum operational lifetime of the biosensor cannot yet be established.

**Figure 6:**
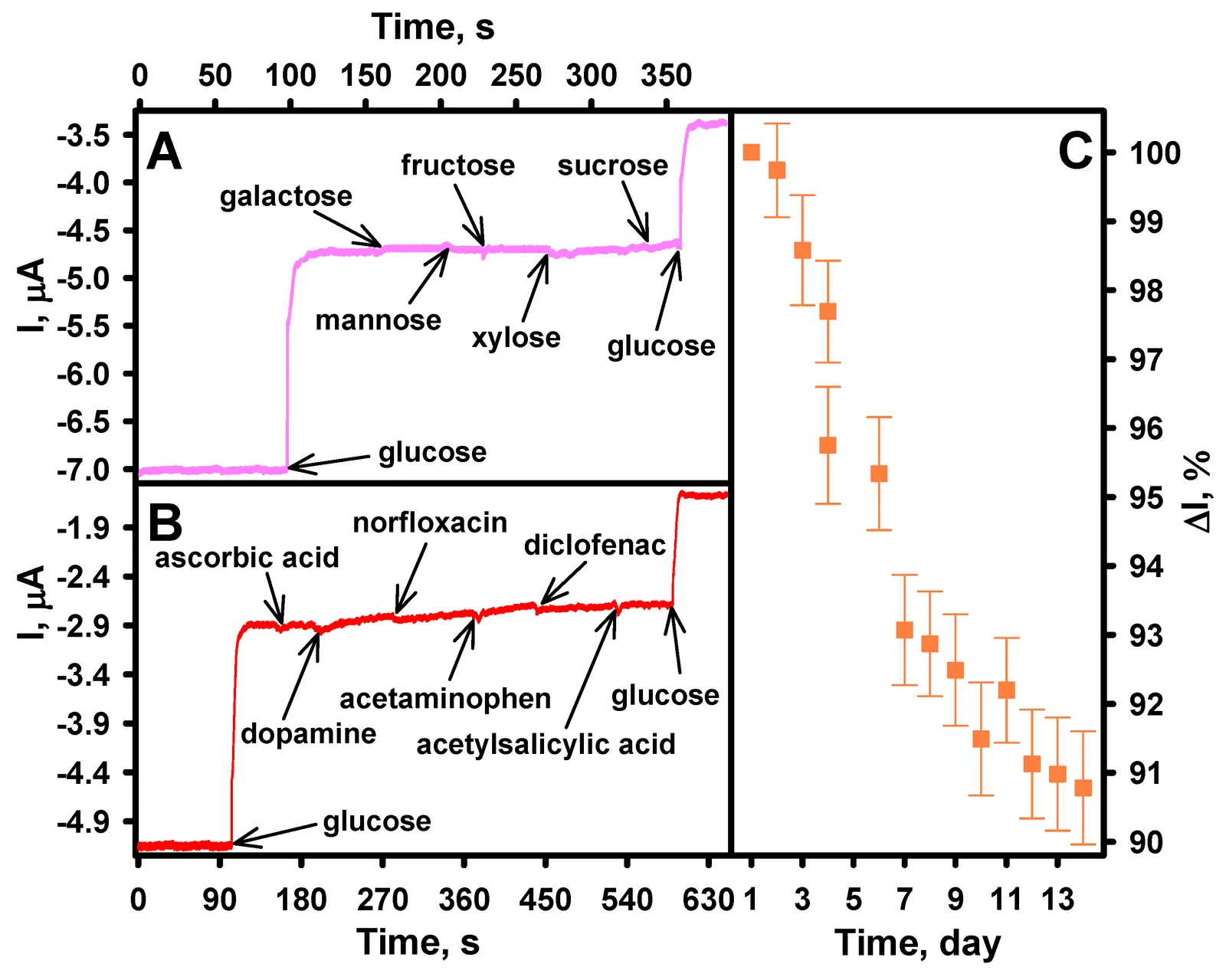
Current response of the biosensor to glucose, other saccharides (**A**) and some interfering substances (**B**). The stability of the biosensor (**C**). GR/NiPtPd/GOx/Aquivion^®^ preparation conditions: 3.0 µL of NiPtPd; 9.0 µL of GOx; 5.0 µL of 1.0% Aquivion^®^. Measurement conditions: APB-KCl (pH 5.5); +0.10 V applied potential vs. Ag/AgCl_3.0 M KCl_; 1.0 mM of glucose and 5.0 mM of other saccharides (**A**); 1.0 mM of glucose, 0.2 mM of ascorbic acid, 0.1 mM of dopamine, 3.0 µM of norfloxacin, 0.2 mM of acetaminofen, 0.2 mM of diclofenac, and 0.2 mM of acetylsalicylic acid (**B**); 1.0 mM of glucose (**C**). Error bars represent the standard deviation of three independently prepared electrodes (n = 3).

The selectivity of the developed biosensor toward glucose was evaluated by evaluating its amperometric response in the presence of structurally related saccharides, including galactose, mannose, fructose, xylose, and sucrose. As shown in Figure 6A, the addition of these compounds produced negligible changes in the amperometric signal, whereas a pronounced response was observed following glucose addition. These results demonstrate that the biosensor exhibits high selectivity toward glucose under the selected measurement conditions. The observed selectivity can be primarily attributed to the substrate specificity of GOx, which efficiently catalyzes the oxidation of glucose while exhibiting little or no activity toward the tested saccharides [49,50]. In addition, the absence of significant responses to the interfering sugars indicates that the incorporation of NiPtPd nanoparticles does not adversely affect the selectivity of the enzymatic recognition process. Therefore, the developed GR/NiPtPd/GOx/Aquivion^®^-based biosensor shows good potential for selective glucose determination in samples containing other common saccharides.

Real glucose-containing samples usually comprise not only various saccharides but also electroactive endogenous and exogenous species that, at sufficiently applied potentials, can undergo oxidation or reduction at the working electrode surface and thereby interfere with the amperometric response of the biosensor. Consequently, resistance to interference represents an important requirement for the practical application of electrochemical glucose biosensors [37]. To evaluate the anti-interference ability of the developed biosensor toward common electroactive species, the influence of ascorbic acid, dopamine, norfloxacin, acetaminophen, diclofenac, and acetylsalicylic acid was investigated. As shown in Figure 6B, the addition of glucose produced a pronounced increase in the current response, whereas the tested interfering substances caused only negligible signal changes or no measurable response, even when added at concentrations comparable to or higher than those typically found in real samples. For example, the physiological or therapeutic concentration ranges of ascorbic acid, acetaminophen, and acetylsalicylic acid are approximately 11–50 µM, 33–166 µM, and 0.6–1.7 mM [38], respectively. Notably, subsequent addition of glucose again generated a distinct current increase, confirming that the biosensor retained its responsiveness in the presence of the investigated interferents. The observed resistance toward interference can be attributed to the low operating potential (+0.10 V) as well as to the presence of the Aquivion^®^ polymer layer on the GR/NiPtPd/GOx/Aquivion^®^ electrode surface. Similar to Nafion [37,51], Aquivion^®^ contains negatively charged sulfonate groups that can electrostatically repel negatively charged interfering species from the electrode surface, thereby reducing their contribution to the measured current. As a result, the developed biosensor exhibited high selectivity toward glucose even in the presence of commonly encountered electroactive compounds.

The storage stability of the developed biosensor was evaluated by periodically measuring its amperometric response to 1.0 mM glucose over a period of 14 days. Between measurements, the GR/NiPtPd/GOx/Aquivion^®^ electrode was stored in a humid environment at +4 °C. As shown in Figure 6C, the biosensor exhibited only a gradual decrease in response during the storage period and retained 90.78% of its initial current signal after 14 days. Although the decrease was somewhat more pronounced between days 3 and 6, no sudden loss of activity was observed, and the overall decrease remained moderate throughout the storage period. The high signal retention demonstrates good short-term storage stability of the developed biosensor and indicates that the sensing layer remains largely functional under the applied storage conditions. Similar short-term stability has been reported for other GOx-based biosensors, although direct comparison is difficult because storage performance strongly depends on the immobilization strategy, sensing matrix, and storage conditions. Previous studies have shown that GOx-based biosensors may retain stable responses for several weeks, while gradual losses in sensitivity are commonly observed during prolonged storage over several months [52,53]. The present results therefore demonstrate that the GOx–NiPtPd sensing layer maintains its functionality during short-term storage under the conditions employed in this study. It should be noted, however, that the long-term durability of the sensing layer was not evaluated in the present study. During prolonged storage, factors such as gradual GOx deactivation, changes in the hydration state of the sensing layer, exposure to humidity and buffer vapors, and possible alterations of the nanoparticle surface may affect biosensor performance. In addition, operational stability during repeated measurements was not investigated and should be addressed in future studies.

### 3.6. Analysis of Human Serum Samples

The applicability of the developed biosensor to the analysis of real samples was assessed by measuring glucose in human serum. The serum used in this study was commercially obtained, and the study did not involve the recruitment of human subjects or the collection of human samples specifically for this work. A commercially available serum sample, whose glucose concentration was independently estimated using a handheld glucometer, was added to an electrochemical cell containing APB-KCl buffer (pH 5.5) in an amount corresponding to a final glucose concentration of 0.087 mM. Glucose determination was performed in triplicate under identical experimental conditions, and the concentration was quantified using the previously established calibration curve. The obtained results, summarized in Table 3, showed an average recovery of 101.28% with an RSD of 5.27%, indicating good accuracy and precision of the measurements. It should be noted that the handheld glucometer was used only to independently estimate the glucose concentration in the serum sample and served as a comparative rather than a reference analytical method. Although commercial glucometers are designed for measurements under physiological conditions, the serum sample analyzed in this study was introduced into the electrochemical cell as a small aliquot and measured under the same conditions as those used for biosensor calibration. Therefore, the comparison was intended to provide an approximate validation of the obtained results rather than a rigorous method-to-method evaluation. These findings provide preliminary evidence of the applicability of the developed biosensor for glucose determination in diluted serum samples. The satisfactory recovery values further suggest that matrix components present in the analyzed sample did not significantly affect the analytical performance of the biosensor under the employed measurement conditions. Because only a small aliquot of serum was added to the electrochemical cell, the sample matrix was substantially diluted, which likely further reduced the influence of potential interfering components. Therefore, the influence of protein fouling, ionic strength variations, and salting-out phenomena was likely limited under the conditions employed in this study. However, these factors may become more significant in less diluted biological samples. Furthermore, the negatively charged sulfonate groups present in Aquivion^®^ may provide a degree of permselectivity that contributes to the observed anti-interference characteristics of the biosensor.

## 4. Conclusions

In this study, an amperometric glucose biosensor based on the combined use of GOx and trimetallic NiPtPd nanoparticles was successfully developed and systematically evaluated. The NiPtPd nanoparticles exhibited pronounced peroxidase-like activity and enabled efficient electrocatalytic transduction of the enzymatic reaction at low potential. The resulting GR/NiPtPd/GOx/Aquivion^®^ biosensor provided a reliable amperometric response toward glucose, exhibiting a linear range of 0.04–1.58 mM, an LOD of 17.33 µM, and a sensitivity of 29.84 μA/mM cm^2^. The biosensor demonstrated good repeatability, satisfactory reproducibility, good short-term storage stability, and high resistance to common electroactive interferents, owing to its operation at a low applied potential of +0.10 V. Analysis of a diluted human serum sample provided preliminary evidence of the applicability of the developed sensing platform in biological matrices. In addition, the observed amperometric behavior indicates that the biosensor response may be associated primarily with the electroreduction of enzymatically generated hydrogen peroxide, while oxygen consumption during the enzymatic reaction may also influence the measured current response. These observations suggest that coupled enzymatic and electrochemical processes may contribute to signal generation in low-potential oxidase–nanozyme biosensors. Overall, this work demonstrates that trimetallic NiPtPd nanoparticles can serve as efficient low-potential electrocatalytic transducers in enzyme–nanozyme biosensing systems.

## Figures and Tables

**Table 1:** Comparison of the analytical characteristics of the developed biosensor and some previously published amperometric glucose enzyme–nanozyme-based biosensors.

Working Electrode	Applied Potential, V	Linear Range, mM	LOD, µM	LOQ, µM	Sensitivity, μA/mM cm^2^	Ref.
GR/NiPtPd/GOx/Aquivion	+0.1	0.04–1.58	17.33	52.53	29.84	this work
GR/PtCo/GOx/Nafion	−0.3	0.04–2.18	21	64	19.38	[32]
GR/CuHCF/GOx/Nafion	−0.25	up to 0.50	–	–	32.2	[29]
Pt/GOx/Fe3O4/Cs/Nafion	+0.4	0.006–2.2	6	–	11.54	[11]
GC/CuWO4/GOx/Nafion	–	0.005–1.8	1.5	–	28.02	[31]
GC/NiFe_2_/OMC/GOx/Nafion	−0.2	0.049–12.5	0.24	–	6.9	[20]
GC/GOx@ZIF-8(TiO_2_)	−0.45	–	0.08	–	–	[30]
ITO/CMC-PPy-PBNPs/GOx	−0.05	0.02–1.1	5.23	–	456.8	[44]
GE/PB/SnO_2_–NF/GOx	−0.1	0.5–5	50	–	3	[48]
Au/rGO/PtPd/GOx	+0.55	2–12	1	–	24	[19]

Cs—chitosan; GC—glassy carbon electrode; OMC—ordered mesoporous carbon; ZIF-8—zeolitic imidazolate framework-8; ITO—indium tin oxide-coated glass electrode; CMC—carboxymethylcellulose; PPy—polypyrrole; PBNPs—Prussian Blue nanoparticles; SnO_2_–NF—multiporous tin oxide nanofiber film; PB—Prussian Blue; rGO—reduced graphene oxide.

**Table 2:** Repeatability and reproducibility of biosensor evaluated at 0.6 mM glucose.

Study	ΔI, µA	Average of ΔI, µA	STDEV	RSD, %
Repeatability	1.43	1.37	0.05	4.01
	1.35			
	1.29			
	1.41			
	1.38			
Reproducibility	1.89	1.66	0.15	9.00
	1.64			
	1.61			
	1.70			
	1.48			

**Table 3:** Data obtained from the determination of glucose concentration in human serum.

Glucose, mM	ΔI, µA	Detected Glucose, mM	Recovery, %	Average, %	RSD, %
0.087	0.33	0.088	101.21	101.28	5.27
0.087	0.34	0.093	106.65		
0.087	0.32	0.084	95.98		

## Data Availability

The data presented in this study are available on request from the first author.

## Abbreviations

The following abbreviations are used in this manuscript:

GOx	Glucose oxidase
NiPtPd	Trimetallic nickel–platinum–palladium
RSD	Relative standard deviation
PO-NZ	Peroxidase-like nanozyme
GR	Graphite rod
UHQ	Ultra-high-quality
APB-KCl	Acetate-phosphate buffer containing KCl
FE-SEM	Field-emission scanning electron microscope
GR/NiPtPd	Graphite rod electrode modified with NiPtPd nanoparticles
GR/NiPtPd/Aquivion^®^	Graphite rod electrode modified with NiPtPd nanoparticles and Aquivion^®^
GR/NiPtPd/GOx	Graphite rod electrode modified with NiPtPd nanoparticles and glucose oxidase
GR/NiPtPd/GOx/Aquivion^®^	graphite rod electrodes modified with NiPtPd nanoparticles, glucose oxidase and Aquivion^®^
ΔI	Change in current
LOD	Limit of detection
LOQ	Limit of quantification
PtCo	Bimetallic platinum–cobalt nanoparticles
Cs	Chitosan
GC	Glassy carbon electrode
OMC	Ordered mesoporous carbon
ZIF-8	Zeolitic imidazolate framework-8
ITO	Indium tin oxide-coated glass electrode
CMC	Carboxymethylcellulose
PPy	Polypyrrole
PBNPs	Prussian Blue nanoparticles
SnO_2_–NF	Multiporous tin oxide nanofiber film
PB	Prussian Blue
rGO	Reduced graphene oxide
STDEV	Standard deviation

## References

[1] Clark L.C., Lyons C. (1962). Electrode systems for continuous monitoring in cardiovascular surgery. Ann. N. Y. Acad. Sci..

[2] Singh R., Nema P., Purohit A., Vishwakarma S., Tantuway S., Namdeo P., Kumar A., Soni V., Kashaw S.K. (2026). Advancements in glucose biosensors: Innovations for wearable and real-time monitoring. Talanta.

[3] Guan G., Qu L., Zhao Y., Wang P., Kong F., Zhang Y., Lin Z., Ni X., Zhang X., Lu Q. (2025). Recent advances in glucose monitoring utilizing oxidase electrochemical biosensors integrating carbon-based nanomaterials and smart enzyme design. Front. Chem..

[4] Jia W.-Z., Wang K., Xia X.-H. (2010). Elimination of electrochemical interferences in glucose biosensors. TrAC Trends Anal. Chem..

[5] Shamsabadi A., Haghighi T., Carvalho S., Frenette L.C., Stevens M.M. (2024). The nanozyme revolution: Enhancing the performance of medical biosensing platforms. Adv. Mater..

[6] Cai X., Huang Y., Zhu C. (2025). Immobilized multi-enzyme/nanozyme biomimetic cascade catalysis for biosensing applications. Adv. Healthc. Mater..

[7] Tan R., Jiang P., Gao N., Cai Z., Grushevskaya H., Wu Y., He H., He Y., Chang G. (2025). Bioactive metallic nanozymes-based electrochemical biosensors for biomarker detection: Progress and prospects. Nano Mater. Sci..

[8] Demkiv O., Nogala W., Stasyuk N., Grynchyshyn N., Vus B., Gonchar M. (2023). The peroxidase-like nanocomposites as hydrogen peroxide-sensitive elements in cholesterol oxidase-based biosensors for cholesterol assay. J. Funct. Biomater..

[9] Ko E., Tran V.-K., Son S.E., Hur W., Choi H., Seong G.H. (2019). Characterization of Au@PtNP/GO nanozyme and its application to electrochemical microfluidic devices for quantification of hydrogen peroxide. Sens. Actuators B.

[10] Kader M.A., Azmi N.S., Kafi A.K.M., Hossain M.S., Jose R., Goh K.W. (2023). Ultrasensitive nonenzymatic real-time hydrogen peroxide monitoring using gold nanoparticle-decorated titanium dioxide nanotube electrodes. Biosensors.

[11] Yang L., Ren X., Tang F., Zhang L. (2009). A practical glucose biosensor based on Fe_3_O_4_ nanoparticles and chitosan/nafion composite film. Biosens. Bioelectron..

[12] Weng X., Li M., Chen L., Peng B., Jiang H. (2024). A wearable nanozyme–enzyme electrochemical biosensor for sweat lactate monitoring. Talanta.

[13] Tong L., Wu L., Su E., Li Y., Gu N. (2023). Recent advances in the application of nanozymes in amperometric sensors: A review. Chemosensors.

[14] Kurup C.P., Ahmed M.U. (2023). Nanozymes towards personalized diagnostics: A recent progress in biosensing. Biosensors.

[15] Nemati S.S., Dehghan G., Rashtbari S., Tan T.N., Khataee A. (2023). Enzyme-based and enzyme-free metal-based glucose biosensors: Classification and recent advances. Microchem. J..

[16] Juska V.B., Pemble M.E. (2020). A Critical review of electrochemical glucose sensing: Evolution of biosensor platforms based on advanced nanosystems. Sensors.

[17] Nor N.M., Ridhuan N.S., Razak K.A. (2022). Progress of enzymatic and non-enzymatic electrochemical glucose biosensor based on nanomaterial-modified electrode. Biosensors.

[18] Tran H.V., Le T.A., Giang B.L., Piro B., Tran L.D. (2019). Silver nanoparticles on graphene quantum dots as nanozyme for efficient H_2_O_2_ reduction in a glucose biosensor. Mater. Res. Express.

[19] Hossain M.F., Park J.Y. (2014). Amperometric glucose biosensor based on Pt-Pd nanoparticles supported by reduced graphene oxide and integrated with glucose oxidase. Electroanalysis.

[20] Xiang D., Yin L., Ma J., Guo E., Li Q., Li Z., Liu K. (2015). Amperometric hydrogen peroxide and glucose biosensor based on NiFe_2_/ordered mesoporous carbon nanocomposites. Analyst.

[21] Wang Z., Zhang R., Yan X., Fan K. (2020). Structure and activity of nanozymes: Inspirations for de novo design of nanozymes. Mater. Today.

[22] Fan J., Yin J.J., Ning B., Wu X., Hu Y., Ferrari M., Anderson G.J., Wei J., Zhao Y., Nie G. (2011). Direct evidence for catalase and peroxidase activities of ferritin-platinum nanoparticles. Biomaterials.

[23] Ma M., Zhang Y., Gu N. (2011). Peroxidase-like catalytic activity of cubic Pt nanocrystals. Colloids Surf. A Physicochem. Eng. Asp..

[24] Ding Y., Yang B., Liu H., Liu Z., Zhang X., Zheng X., Liu Q. (2018). FePt-Au ternary metallic nanoparticles with the enhanced peroxidase-like activity for ultrafast colorimetric detection of H_2_O_2_. Sens. Actuators B.

[25] Stasyuk N., Gayda G., Demkiv O., Darmohray L., Gonchar M., Nisnevitch M. (2021). Amperometric biosensors for L-arginine determination based on L-arginine oxidase and peroxidase-like nanozymes. Appl. Sci..

[26] Peng F.F., Zhang Y., Gu N. (2008). Size-dependent peroxidase-like catalytic activity of Fe_3_O_4_ nanoparticles. Chin. Chem. Lett..

[27] Zhang K., Zuo W., Wang Z., Liu J., Li T., Wang B., Yang Z. (2015). A simple route to CoFe_2_O_4_ nanoparticles with shape and size control and their tunable peroxidase-like activity. RSC Adv..

[28] Chen L., Kätelhön E., Compton R.G. (2020). Particle-modified electrodes: General mass transport theory, experimental validation, and the role of electrostatics. Appl. Mater. Today.

[29] Gayda G.Z., Demkiv O.M., Gurianov Y., Serkiz R.Y., Klepach H.M., Gonchar M.V., Nisnevitch M. (2021). “Green” Prussian blue analogues as peroxidase mimetics for amperometric sensing and biosensing. Biosensors.

[30] Paul A., Srivastava D.N. (2018). Amperometric glucose sensing at nanomolar level using MOF-encapsulated TiO_2_ platform. ACS Omega.

[31] Zhang Y., Li S., Liu H., Shi F., Li J., Hub X., Yang Z. (2022). Dual-strategy biosensing of glucose based on multifunctional CuWO_4_ nanoparticles. Analyst.

[32] Kausaite-Minkstimiene A., Krikstaponyte A., Stasyuk N., Gayda G., Ramanaviciene A. (2025). An amperometric enzyme–nanozyme biosensor for glucose detection. Biosensors.

[33] Bankar S.B., Bule M.V., Singhal R.S., Ananthanarayan L. (2009). Glucose oxidase—An overview. Biotechnol. Adv..

[34] Demkiv O., Gayda G., Stasyuk N., Moroz A., Serkiz R., Kausaite-Minkstimiene A., Gonchar M., Nisnevitch M. (2023). Flavocytochrome b_2_-mediated electroactive nanoparticles for developing amperometric L-lactate biosensors. Biosensors.

[35] Demkiv O., Gayda G., Stasyuk N., Brahinetz O., Gonchar M., Nisnevitch M. (2022). Nanomaterials as redox mediators in laccase-based amperometric biosensors for catechol assay. Biosensors.

[36] Gayda G., Demkiv O., Stasyuk N., Klepach H., Serkiz R., Nakonechny F., Gonchar M., Nisnevitch M. (2025). Copper hexacyanoferrates obtained via flavocytochrome b_2_ assistance: Characterization and application. Biosensors.

[37] Maalouf R., Chebib H., Saïkali Y., Vittori O., Sigaud M., Jaffrezic-Renault N. (2007). Amperometric and impedimetric characterization of a glutamate biosensor based on Nafion^®^ and a methyl viologen modified glassy carbon electrode. Biosens. Bioelectron..

[38] Isailović J., Dapčević A., Žunić M., Finšgar M., Vidović K., Tasić N., Hočevar S.B. (2025). Study of a sensitive and selective electrochemical biosensor for glucose based on Bi_2_Ru_2_O_7_ pyrochlore clusters combined with MWCNTs. Chemosensors.

[39] Tsai Y.-C., Li S.-C., Chen J.-M. (2005). Cast thin film biosensor design based on a nafion backbone, a multiwalled carbon nanotube conduit, and a glucose oxidase function. Langmuir.

[40] Liu Y., Nan X., Shi W., Liu X., He Z., Sun Y., Ge D. (2019). A glucose biosensor based on the immobilization of glucose oxidase and Au nanocomposites with polynorepinephrine. RSC Adv..

[41] Wijayanti S.D., Tsvik L., Haltrich D. (2023). Recent advances in electrochemical enzyme-based biosensors for food and beverage analysis. Foods.

[42] Vokhmyanina D.V., Andreeva K.D., Komkova M.A., Karyakina E.E., Karyakin A.A. (2020). ‘Artificial peroxidase’ nanozyme–enzyme based lactate biosensor. Talanta.

[43] Das B., Franco J.L., Logan N., Balasubramanian P., Kim M.I., Cao C. (2021). Nanozymes in point-of-care diagnosis: An emerging futuristic approach for biosensing. Nano Micro Lett..

[44] Uzunçar S., Özdoğan N., Ak M. (2021). Amperometric detection of glucose and H_2_O_2_ using peroxide selective electrode based on carboxymethylcellulose/polypyrrole and Prussian Blue nanocomposite. Mater. Today Commun..

[45] Wong C.M., Wong K.H., Chen X.D. (2008). Glucose oxidase: Natural occurrence, function, properties and industrial applications. Appl. Microbiol. Biotechnol..

[46] Ferreira L.F.P., Taqueda M.E., Converti A., Vitolo M., Pessoa A. (2005). Purification of glucose oxidase from *Aspergillus niger* by liquid-liquid cationic reversed micelles extraction. Biotechnol. Prog..

[47] Jv Y., Li B., Cao R. (2010). Positively-charged gold nanoparticles as peroxidiase mimic and their application in hydrogen peroxide and glucose detection. Chem. Commun..

[48] Kafi A.K.M., Alim S., Jose R., Yusoff M.M. (2019). Fabrication of a glucose oxidase/multiporous tin-oxide nanofiber film on Prussian blue–modified gold electrode for biosensing. J. Electroanal. Chem..

[49] Adams E.C., Mast R.L., Free A.H. (1960). Specificity of glucose oxidase. Arch. Biochem. Biophys..

[50] Kornecki J.F., Carballares D., Tardioli P.W., Rodrigues R.C., Berenguer-Murcia Á., Alcántara A.R., Fernandez-Lafuente R. (2020). Enzyme production of D-gluconic acid and glucose oxidase: Successful tales of cascade reactions. Catal. Sci. Technol..

[51] Ghannouchi S., Özer E.M., Çamurlu P. (2025). New amperometric glucose biosensors based on Nafion nanofibers. Sci. Res. Commun..

[52] Chmayssem A., Shalayel I., Marinesco S., Zebda A. (2023). Investigation of GOx Stability in a chitosan matrix: Applications for enzymatic electrodes. Sensors.

[53] Jędrzak A., Kuznowicz M., Jesionowski T. (2024). Mobile-assisted diagnostic biosensor for point-of-care glucose detection in real human samples with rapid response and long-live stability. J. Appl. Electrochem..

